# The importance of the intensive care unit environment in sleep—A study with healthy participants

**DOI:** 10.1111/jsr.12959

**Published:** 2019-12-13

**Authors:** Laurens Reinke, Marjolein Haveman, Sandra Horsten, Thomas Falck, Esther M. van der Heide, Sander Pastoor, Johannes H. van der Hoeven, Anthony R. Absalom, Jaap E. Tulleken

**Affiliations:** ^1^ Department of Critical Care University Medical Center Groningen University of Groningen Groningen the Netherlands; ^2^ Philips Research Eindhoven the Netherlands; ^3^ Department of Neurology University Medical Center Groningen University of Groningen Groningen the Netherlands; ^4^ Department of Anaesthesiology University Medical Center Groningen University of Groningen Groningen the Netherlands

**Keywords:** critical illness, first‐night effect, polysomnography

## Abstract

Sleep disruption is common among intensive care unit patients, with potentially detrimental consequences. Environmental factors are thought to play a central role in ICU sleep disruption, and so it is unclear why environmental interventions have shown limited improvements in objectively assessed sleep. In critically ill patients, it is difficult to isolate the influence of environmental factors from the varying contributions of non‐environmental factors. We thus investigated the effects of the ICU environment on self‐reported and objective sleep quality in 10 healthy nurses and doctors with no history of sleep pathology or current or past ICU employment participated. Their sleep at home, in an unfamiliar environment (‘Control’), and in an active ICU (‘ICU’) was evaluated using polysomnography and the Richard‐Campbell Sleep Questionnaire. Environmental sound, light and temperature exposure were measured continuously. We found that the control and ICU environment were noisier and warmer, but not darker than the home environment. Sleep on the ICU was perceived as qualitatively worse than in the home and control environment, despite relatively modest effects on polysomnography parameters compared with home sleep: mean total sleep times were reduced by 48 min, mean rapid eye movement sleep latency increased by 45 min, and the arousal index increased by 9. Arousability to an awake state by sound was similar. Our results suggest that the ICU environment plays a significant but partial role in objectively assessed ICU sleep impairment in patients, which may explain the limited improvement of objectively assessed sleep after environmental interventions.

**Abbreviations**
dB(A): A‐weighted sound pressure in decibels
EEG: electroencephalogram
EMG: electromyogram
EOG: electrooculogram
FNE: first‐night effect
KSS: Karolinska Sleepiness Scale
LAeq: A‐weighted per‐second sound level
PSG: polysomnography
REM: rapid eye movement
RR_arousal_: relative risk for an arousal
SFI: sleep fragmentation index
SPF: Samn−Perelli Fatigue
SWS: slow‐wave sleep
TST: total sleep time

## 1 INTRODUCTION

The biological function of sleep is not fully understood, even though sleep is known to be essential for human homeostasis and survival (Kamdar, Needham, & Collop, 2012). Unfortunately, sleep disruption is common in the hospital setting, especially in the intensive care unit (ICU; Hilton, 1976; Xie, Kang, & Mills, 2009). Most ICU patients exhibit severely disturbed sleeping patterns, characterized by severe fragmentation by frequent arousals and awakenings (Andersen, Boesen, & Olsen, 2013; Bourne, Minelli, Mills, & Kandler, 2007; Friese, Diaz‐Arrastia, McBride, Frankel, & Gentilello, 2007). Furthermore, their sleep generally lacks slow‐wave sleep (SWS) and rapid eye movement (REM) sleep stages (Boyko, Ording, & Jennum, 2012). This may increase their susceptibility to infections (Boyko et al., 2012; Cooper, 2000; Friese et al., 2007), lead to alterations in wound healing (Cooper et al., 2000; Friese et al., 2007), and impaired neurophysiological organization and memory consolidation (Boyko et al., 2012), which in turn may lead to the development of delirium, prolonged admission and increased mortality risk among ICU patients (Boyko et al., 2012).

The aetiology of ICU sleep disruption is not well understood, although it is commonly thought to be caused by environmental factors in addition to influences from the underlying illness, medication, sedation, mechanical ventilation and other discomforts as a result of treatment (Gabor et al., 2003; Kamdar et al., 2012; Xie et al., 2009). A‐weighted ICU noise levels consistently exceed recommended levels (Busch‐Vishniac et al., 2005; MacKenzie & Galbrun, 2007; Pulak & Jensen, 2016; Tegnestedt et al., 2013), and are dominated by high‐frequency noise (Darbyshire & Young, 2013) caused by mechanical ventilators, monitor alarms and staff conversations (Xie et al., 2009).

Controlled nocturnal exposure of volunteers to pre‐recorded ICU noise decreases total sleep time (TST), total REM sleep time and sleep efficiency, while increasing REM sleep latency and the incidence of arousals (Freedman, Kotzer, & Schwab, 1999; Topf, 1992). However, noise has only indirectly been linked to sleep disruption in ICU patients, and the differences between patients are not well understood (Aaron et al., 1996; Freedman et al., 1999; Gabor et al., 2003; Xie et al., 2009). Furthermore, these patient studies were hampered by small sample sizes, low quality of evidence and high risks of bias, further limiting the generalizability of their results (Horsten, Reinke, Absalom, & Tulleken, 2018).

Although frequently blamed as the root cause of sleep disruption, noise is likely only part of the problem. Patients in critical care settings generally have limited or no exposure to zeitgebers such as high‐intensity natural light, regular food intake, physical exercise and social interaction (Castro, Angus, & Rosengart, 2011; Giménez et al., 2011; Korompeli et al., 2017; Schaefer, Williams, & Zee, 2012). Artificial lighting is of insufficient intensity, and exposure at night, even at lower intensities, has an adverse effect on sleep timing (Wang & Greenberg, 2013). The thermal environment is also important for human sleep (Lan, Pan, Lian, Huang, & Lin, 2014). Total sleep time and sleep efficiency seem to favour lower temperatures, which may also increase the duration of REM sleep and SWS, although the effects on ICU sleep are unknown (Valham, Sahlin, Stenlund, & Franklin, 2012).

Besides these potentially modifiable sleep disruptors, the unfamiliarity of the environment is also important (Jay, Aisbett, Sprajcer, & Ferguson, 2015). Bruyneel and colleagues found that polysomnography (PSG) performed at home exhibited longer and more efficient sleep than in‐hospital recordings, with shorter sleep latency and more REM sleep (Bruyneel et al., 2011). This phenomenon of suboptimal sleep in new environments is commonly known as the first‐night effect (FNE; Tamaki, Nittono, Hayashi, & Hori, 2005). The FNE is thought to be caused by one hemisphere being more vigilant and acting as a night watch to monitor unfamiliar surroundings during sleep (Tamaki, Bang, Watanabe, & Sasaki, 2016), and is most pronounced during the first night in an unfamiliar environment (Tamaki et al., 2005).

The quality of sleep of ICU patients is therefore likely impacted cumulatively by the underlying critical illness and treatment, the ICU environment, and the arousing effect of an unknown environment (Boyko et al., 2012). Due to simultaneous exposure, which also changes over time and between patients, the interpretation of partially successful interventions is difficult, and the importance of other environmental factors is largely unknown. To be able to lessen the impact of a real ICU environment on sleep, the relative importance of its elements first needs to be determined.

The aim of our study was to quantify the relative contribution of the ICU environment to the quality of sleep in the ICU. By studying healthy participants at home, in the ICU, and in a controlled quiet hospital environment we eliminate the contribution of critical illness and treatment‐related discomforts, while isolating and quantifying most environmental factors that disrupt sleep in a real‐life scenario.

## 2 METHODS

### 2.1 Procedure and participants

Ten healthy nurses and doctors, either qualified or in specialist training, took part in this prospective repeated‐measures crossover pilot between January and March 2017. Exclusion criteria were: current or past employment on an ICU, pre‐existing history or treatment of sleep pathology, use of sleep‐promoting medication, and alcohol addiction or illicit drug abuse. After obtaining informed consent for participation, participants’ hearing abilities were tested using the online hearing test based on the Fletcher−Munson curve of equal loudness (Fletcher & Munson, 1933; Hatsidimitris).

Each participant was monitored on 1 night in each of three locations: (a) at home; (b) on a busy ICU (“ICU”) in a bed between those of critically ill patients; and (c) on an empty ICU (“control”) to act as a control environment to quantify the FNE. For the control environment, a hospital bed in one of two windowless single patient rooms in a temporarily empty nine‐bed ICU was used. All devices in the room and the adjacent empty multi‐bed room were turned off, and participants were not disturbed until the next morning. Participants were free to turn lights on or off. For the ICU measurement night, volunteers slept in the vertex of a V‐shaped 11‐bed ICU in the same hospital, with patients on either side receiving intensive care with the required suite of bedside devices. The study bed was located opposite a glass medication preparation room and facing away from east‐facing windows. Measurement nights were separated by at least 3 days to avoid acclimatization to the measurement setup, and the order of the active and control ICU measurement nights was randomized for the same reason (Figure S1). The local medical ethics committee reviewed and approved the study protocols (research project number 2016‐647). The study was registered in the online Dutch Trial Register (NTR6189).

### 2.2 Sleep

Polysomnographic sleep recording included a six‐channel electroencephalogram (EEG), two‐channel electrooculogram (EOG) and an electromyogram (EMG) of the left and right masseter muscle or the submental muscles. EEG‐electrodes were placed according to the international 10–20 system with Ag/AgCl electrodes with a common reference. Patients' skin was prepared according to standard techniques. During ambulatory home measurements, the EEG, EMG and EOG were sampled at 256 Hz using either an Embla^®^ A10 (Medcare) or Morpheus^®^ (Micromed) digital recorder. Analogue ICU sleep data were digitized at 500 Hz and recorded electronically using a Alice 6 LDx system (Philips Respironics). A trained neurologist with extensive experience with all three PSG systems visually scored all overnight PSG recordings using standard AASM rules based on Rechtschaffen & Kales criteria, in 30‐s epochs (Rechtschaffen & Kales, 1969). Because arousal scoring criteria are generally well defined, they were annotated by the clinically validated Somnolyzer 24 × 7 sleep scoring software (Philips Respironics), minimizing workload and increasing the comparability within the sample (Punjabi et al., 2015).

Volunteers self‐evaluated sleep quality, sleepiness and fatigue after each night using the six‐item Richard‐Campbell Sleep Questionnaire (RCSQ; Richards, O'Sullivan, & Phillips, 2000), Karolinska Sleepiness Scale (KSS; Akerstedt & Gillberg, 1990) and Samn−Perelli Fatigue (SPF) scale (Samn & Perelli, 1982), respectively. The mean of the first five items of the RSCQ was used as the overall sleep score. Participants did not take naps before the measurement nights.

The sleep period was defined as the time from the moment when the lights were switched off until the moment the participant rose from bed in the morning, as documented in a sleep diary. Sleep efficiency was defined as the fraction of sleep during the sleep period. Sleep latency was defined as the time between lights off and the first epoch of sleep. Lights off time was derived manually from Actiwatch Spectrum (Philips Respironics) luminance data. Awakenings were defined as transitions to the wake stage after sleep onset. The sleep fragmentation index (SFI) was calculated by dividing the number of transitions to awake or stage N1 sleep by the TST.

Participants were not allowed to drink caffeine from 12:00 a.m. on the day of the measurements. Also, participants were discouraged to schedule a day or night shift on the day following the measurement.

### 2.3 Sound

For the home baseline measurement, the Philips VitalMinds light and sound assessment application (Philips) was used to store data at 1 Hz. For detailed sound level monitoring in the ICU, an Earthworks M23 microphone (Earthworks) was used. Sound data from the ICU recordings were stored at 18 Hz. The microphone was calibrated before the start of the measurements and placed approximately 1 m above the participant's head. Several recordings were made with both measurement systems simultaneously to detect differences in sensitivity, which were corrected before analysis. A‐weighting was applied to all sound data to mimic the noise response curve of human hearing. The median sound pressure was calculated for 1‐s windows. Arousal analysis focused on the relative risk of an arousal occurring within a 30‐s epoch that contained significant changes in the volume of sound. If an increase of 6 dB(A), i.e. a doubling of the sound amplitude, was found during an epoch of sleep, it was considered significantly noisy. The relative risk was defined as the ratio between the risks of an arousal during an epoch with and without significant noise, respectively.

### 2.4 Temperature

For temperature measurements the Ebro EBI 300 digital environmental USB‐temperature logger (Ebro Electronic GmbH) was used.

### 2.5 Statistical analysis

The sample size was chosen pragmatically, as there was insufficient published data on which to base a formal sample size calculation. All data were processed in Matlab 2016b (Mathworks^®^), statistical analyses were performed in SPSS 23 (IBM). Randomly missing disjoint temperature data (two cases) and sound data (two cases) in the home environment were estimated by mean substitution. A repeated‐measures ANOVA was done to test for within‐subject differences for individual parameters. For parameters that violated Mauchly's test for sphericity, the Greenhouse−Geisser correction was applied. An additional Bonferroni‐adjusted pairwise comparison was made between individual measurement nights.

## 3 RESULTS

Seven qualified nurses, one nurse trainee, a medical intern and a resident participated in the study. Of the 10 participants, nine were female and the average age was 31.9 (11.9) years.

### 3.1 Environmental factors

**Figure 1:**
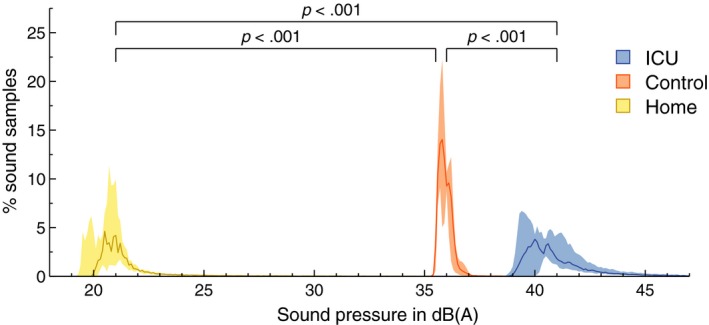
Distribution of sound pressure for home, control and ICU environment. Bold lines indicate the median percentage of all per second sound samples distributed over 0.1‐dB(A)‐wide bins. The interquartile range of this parameter is shaded. The home environment was characterized by a majority of samples in the 19−24 dB(A) range, where the control environment had a much narrower distribution focused between 35 and 37 dB(A). The ICU environment exhibited a wider distribution of sound, with most sound exceeding 39 dB(A)

**Figure 2:**
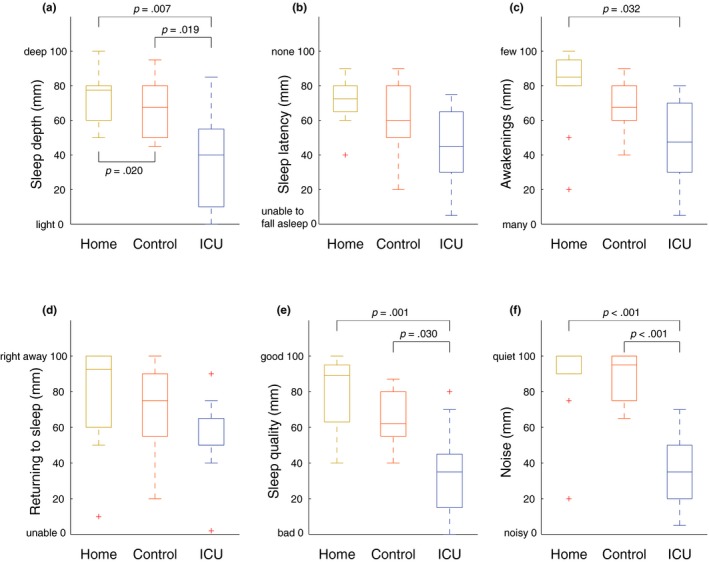
Self‐reported sleep quality. The perceived depth of sleep got progressively worse when transitioning from the home environment through the control environment to the ICU (a). Perceived sleep latency (b) did not differ between study nights. Participants reported significantly more awakenings in the ICU compared with the home environment (c), although they reported similar ease of returning to sleep afterwards (d). The overall perceived quality of sleep (e) and the amount of environmental noise (f) were significantly worse in the ICU compared with the control and home environment

The intensity of ambient light was similar between the environments. Temperature was particularly low in some of the participants’ home environments, which led to significant differences between study nights, as shown in Table 1. Repeated‐measures ANOVA showed that the home environment was more than 5°C colder than the climate‐controlled ICU and control environment. The amount and power distribution of noise between lights off and lights on differed significantly between study nights, as shown in Figure 1. The ICU was significantly more noisy than the control environment, which in turn was significantly more noisy than the home environment. Participants perceived the ICU to be significantly more noisy than the control and home environment, as shown in Figure 2f.

**Table 1:** Environmental factors and sleep quality outcomes

Variables	Home	Control	ICU	*F*	*p‐*value
Total sleep score (mean of RCSQ items 1–5)	76.42 (14.27)	65.90 (8.47)	43.26 (22.29)	7.214	< .002^a^
SPF	3.90 (1.20)	3.70 (1.25)	3.95 (1.34)	0.159	.736
KSS	6.05 (1.34)	6.00 (1.41)	5.65 (2.06)	0.437	.572
Light; lux	0.96 (2.54)	0.81 (1.56)	0.49 (0.67)	0.250	.781
median LAeq; dB(A)	20.74 (0.51)	35.63 (1.46)	41.08 (0.91)	1,063.399	< .001^a^
Temp.; °C	16.51 (3.65)	21.92 (0.38)	21.90 (2.09)	13.144	.003^a^
TST; min	447.20 (46.44)	452.10 (27.10)	404.45 (38.03)	4.986	.019^a^
Sleep efficiency; %	91.73 (4.23)	88.84 (7.66)	84.77 (10.89)	1.835	.188
Sleep latency; min	20.41 (24.23)	27.74 (35.83)	34.14 (39.15)	0.497	.617
REM latency; min	107.25 (58.89)	108.70 (33.71)	154.15 (67.04)	3.888	.039^a^
REM; %	22.00 (8.39)	23.68 (6.30)	19.11 (4.43)	3.561	.050^a^
N1; %	1.85 (1.48)	2.48 (1.87)	3.30 (2.19)	1.488	.252
N2; %	46.55 (5.98)	46.56 (6.47)	54.54 (7.88)	15.799	< .001^a^
N3; %	29.61 (5.08)	27.28 (5.35)	23.05 (4.27)	4.464	.027^a^
Wake after sleep onset; min	35.25 (20.65)	42.30 (22.79)	82.40 (46.87)	6.112	.024^a^
Awakenings per night	21.50 (10.12)	15.10 (11.19)	23.00 (9.76)	2.524	.108
Mean duration of awakenings; min	1.10 (0.28)	1.17 (0.37)	1.81 (0.77)	7.376	.017^a^
Arousal index	6.79 (5.06)	10.49 (3.32)	15.77 (6.06)	8.564	.002^a^
RR_arousal_	1.42 (0.65)	9.59 (5.85)	1.79 (0.71)	12.937	< .001^a^

Note

Data are presented as the mean (*SD*). *p*‐values are calculated using repeated‐measures ANOVA. Non‐spherical measures are corrected using Greenhouse−Geisser to reduce type I error rate.

KSS, Karolinska Sleepiness Scale; LAeq, A‐weighted per second sound level; RCSQ, Richard‐Campbell Sleep Questionnaire; REM, rapid eye movement sleep; RR_arousal_, relative risk of arousal after ΔdB > 6; SPF, Samn−Perelli Fatigue; TST, total sleep time.

a Significant *p*‐values are highlighted.

### 3.2 Self‐reported sleep parameters

Perceived quality of sleep was strongly dependent on the sleeping environment, as shown in Figure 2. Participants reported experiencing significantly lower depth of sleep in the control environment and the ICU, and lower general sleep quality during their night of sleep in the ICU compared with both the home and control night. The participants also reported more awakenings in the ICU compared with the night at home. Self‐reported sleepiness and fatigue scores did not differ significantly between the three study nights (Table S1).

### 3.3 Objective sleep parameters

The objective measures of sleep architecture and duration are summarized in Table 1. Pairwise comparisons between measurement nights are summarized in Table S2in the supplemental material. The mean difference in TST between ICU and control environment was more than 47 min. Repeated‐measures ANOVA revealed significant differences in the distribution of REM, N2 and N3 sleep between the measurement nights. There was a small but significant difference in the percentage of N2 sleep between the home environment and the ICU environment, and between the control environment and the ICU environment. REM latency increased by almost 47 min in the ICU compared with the night at home.

**Figure 3:**
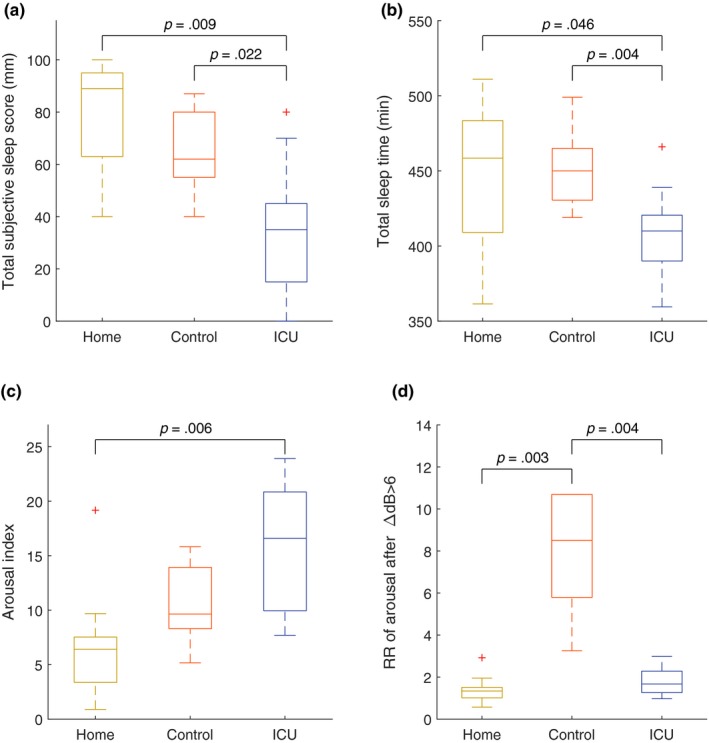
Quality of sleep, awakenings, arousals and arousability. Total perceived sleep score (a) and total sleep time (b) were lowest during a night in the ICU, and significantly lower than in both the control and home environment. Inversely, the arousal index was significantly higher in the ICU than the home environment (c). The relative risk of arousals after changes in sound pressure was significantly higher in the control environment than in the home and ICU environment (d)

Automated arousal scoring showed no significant increase of arousals when sleeping in the control environment relative to the home environment, as shown in Figure 3c. Subjects experienced more arousals during sleep in the ICU environment than during sleep in the home environment. Additionally, the relative risk to experience an arousal after an increase in environmental sound was more than five times higher in the control environment than in the home and ICU environment (Figure 3d).

## 4 DISCUSSION

To our knowledge this is the first study to assess quality of sleep both subjectively and objectively in healthy participants exposed to a real ICU environment, relative to their normal sleeping patterns at home and in a quiet ICU environment. Despite the limited scope, our findings seem to suggest that objective and perceived quality of sleep are impacted differently by not sleeping at home and by sleeping in a noisy environment. Although significant differences in commonly used estimates of quality of sleep were found, none of the participants exhibited disruption of EEG patterns close to the degree observed during the first night of ICU admission of critically ill patients (Elliott, McKinley, & Cistulli, 2011).

The sound measurement results of the current study show that our ICU may not be as noisy as other ICUs reported in past publications (Horsten et al., 2018). There are several potential reasons for this. The first is the possibility of the Hawthorne effect. The staff were aware of the study and may have altered their behaviour by moderating the volume and extent of conversations in the presence of patients, or by early silencing or muting of alarms. We did not, however, find any differences in environmental light and sound on the same ICU before, during and after the experiments. Secondly, our ICU design and layout, patient mix, intensity and number of interventions, and our type and number of monitoring and therapeutic devices emitting sound at night may be different to that of other ICUs.

Gabor and colleagues found that healthy participants exhibited a higher percentage of arousals and awakenings associated with elevations in environmental noise in an open ICU than in a single room (Gabor et al., 2003). Similar to the study of Gabor, our participants experienced high but varying numbers of noise peaks in all environments, due to the relatively low background noise levels. We decided to take the chance occurrence of arousals and noise into account by calculating the relative risk of arousals during an epoch with significant sound increases instead of calculating the absolute percentage of arousals after an increase in sound as Gabor and colleagues did. This approach resulted in a similar arousability between the home and ICU environment.

A possible explanation of the low relative risk for arousals by noise in the ICU is the high level of background noise, and the decreased TST. In the face of overwhelming amounts of noise, it is possible participants were more likely to wake up or stay awake, than to stay asleep and exhibit EEG criteria for arousals. Alternatively, other arousing factors than sound were relatively more common on the ICU than in the control environment. Participants also reported finding the lack of noise and the absence of staff in the empty ICU rather unnerving, which may have further increased their arousability. Finally, it might be the case that exposure to continuous high levels of sound pressure result in a degree of habituation, making volunteers less susceptible to arousal in response to sound peaks.

The arousability by noise was most pronounced in the control environment, supporting the theoretical contribution of the FNE in sleep disruption. The tendency for participants to exhibit increased N2 at the cost of REM is likely the result of increased REM latency and increased arousal incidence.

Our study has some limitations. Firstly, during the ICU measurement the volunteers were not exposed to common ICU discomforts, such as urinary, venous and arterial catheters, endotracheal tubes, thirst, immobility, etc. While a limitation, this is also a strength, as it enables an analysis of the influence of purely environmental factors. Secondly, our study participants all had some experience with the ICU, prior to sleeping on it. This choice was deemed necessary for ethical and safety reasons, but may have moderated the FNE. Thirdly, the small sample size, gender imbalance and relatively young age of the participants limit the statistical power of the study. Interestingly, women are generally more sensitive to sound than men, and young women more sensitive than older women (Pearson et al., 1995). The observed limited effects of environmental noise on objective quality of sleep may therefore overestimate the effects compared with the generally older, more gender‐balanced ICU population.

In conclusion, we found clear signs of sleep disruption in a small group of healthy participants exposed to an ICU environment. This level of disruption exceeded the already adverse FNEs of sleeping in a nearly optimal clinical environment, represented by a closed‐off ICU. Sleep disruption in our healthy participants was less severe than that often seen in critically ill patients, however. This indicates that the role of ICU environmental factors, although significant, is only partially responsible for the severely disrupted sleep often observed in the critically ill. The effect of the ICU environment was more pronounced for perceived quality of sleep than objectively measured sleep parameters. Thus, although we applaud attempts to limit environmental noise, these attempts should be part of a broader tailored effort to investigate and limit exposure to all sleep disruptive factors, both intrinsic and environmental.

## CONFLICT OF INTEREST

LR received partial funding (paid to institution) from Philips Research Eindhoven for a PhD position at the University Medical Center Groningen. TF, EMH and SP are employed fully by Philips Research Eindhoven. The remaining authors (MH, SH, JHH, ARA and JET) did not have any conflicts of interest to declare. No funding was obtained for the purpose of this study.

## AUTHOR CONTRIBUTIONS

LR and MH drafted the first manuscript, all other authors provided feedback on drafts of the paper. All authors (LR, MH, SH, TF, EMH, SP, JHH, ARA and JET) were equally responsible for the conception of the study. MH and SH were responsible for implementation of the study and enrolled participants. ARA, JET and LR collated the data and analysed results. JHH was solely responsible for the clinical scoring of sleep data. LR and JET provided technical input and coordinated the study. All authors contributed to, read, and approved the final version.
