# Supplementary figures and images for: The importance of the intensive care unit environment in sleep—A study with healthy participants
Source: J Sleep Res. 2019 Dec 13;29(2):e12959. doi: 10.1111/jsr.12959 (PMC7154670; doi:10.1111/jsr.12959)

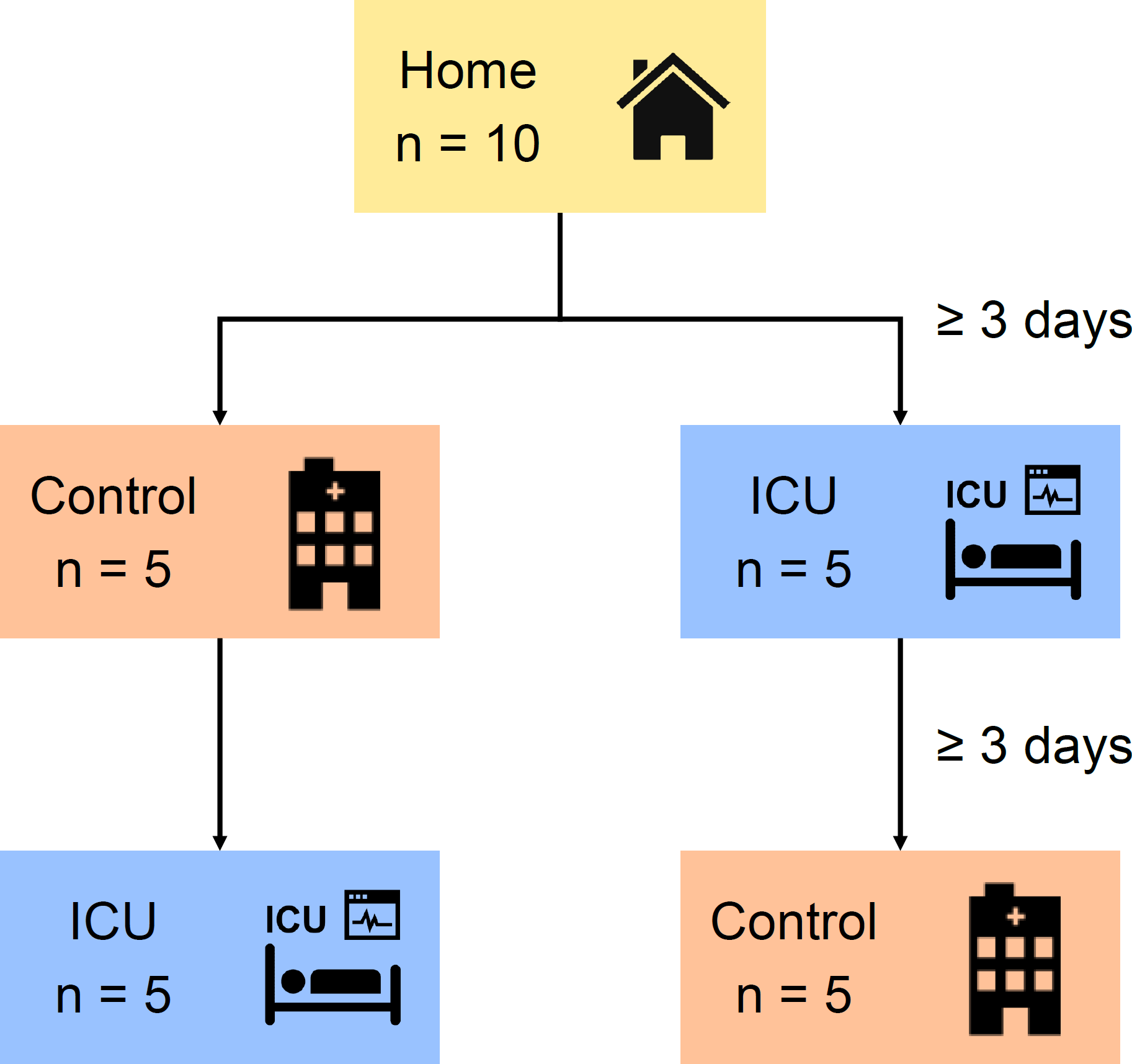

Supplement: Supplementary file 1 [file JSR-29-e12959-s001.tiff]
