# Supplementary material for: The importance of the intensive care unit environment in sleep—A study with healthy participants
Source: J Sleep Res. 2019 Dec 13;29(2):e12959. doi: 10.1111/jsr.12959 (PMC7154670; doi:10.1111/jsr.12959)
Supplement: Supplementary file 2 [file JSR-29-e12959-s002.docx]

**Supplemental Table 1. Pairwise comparison of sleepiness and fatigue**

|  |  |  |  | **95% Confidence interval for difference** | |
| --- | --- | --- | --- | --- | --- |
| **Variables** | **Condition** | **Mean difference (SE)** | **p value** | **Lower bound** | **Upper bound** |
| SPF | Home vs. Control | 0.20 (0.20) | 1.000 | -0.387 | 0.787 |
|  | Home vs. ICU | -0.05 (0.52) | 1.000 | -1.572 | 1.472 |
|  | Control vs. ICU | -0.25 (0.59) | 1.000 | -1.989 | 1.489 |
| KSS | Home vs. Control | 0.05 (0.24) | 1.000 | -0.657 | 0.757 |
|  | Home vs. ICU | 0.40 (0.56) | 1.000 | -1.248 | 2.048 |
|  | Control vs. ICU | 0.35 (0.53) | 1.000 | -1.197 | 1.897 |

SPF = Samn-Perelli Fatigue score, KSS = Karolinska Sleepiness Scale score

Bonferroni adjusted pairwise comparison, with numbers based on estimated marginal means. Data are presented as the mean (SD).
