# Supplementary material for: The importance of the intensive care unit environment in sleep—A study with healthy participants
Source: J Sleep Res. 2019 Dec 13;29(2):e12959. doi: 10.1111/jsr.12959 (PMC7154670; doi:10.1111/jsr.12959)
Supplement: Supplementary file 3 [file JSR-29-e12959-s003.docx]

**Supplemental Table 2. Pairwise comparison of study environments**

|  |  |  |  | **95% Confidence interval for difference** | |
| --- | --- | --- | --- | --- | --- |
| **Variables** | **Condition** | **Mean difference (SE)** | **p value** | **Lower bound** | **Upper bound** |
| Light (lux) | Home vs. Control | 0.15 (0.58) | 1.000 | -1.558 | 1.867 |
|  | Home vs. ICU | 0.47 (0.87) | 1.000 | -2.082 | 3.028 |
|  | Control vs. ICU | 0.32 (0.54) | 1.000 | -1.276 | 1.913 |
| median LAeq (dB) | Home vs. Control | -14.89 (0.48) | <0.001^a^ | -16.311 | -13.468 |
|  | Home vs. ICU | -20.34 (0.33) | <0.001^a^ | -21.302 | -19.367 |
|  | Control vs. ICU | -5.45 (0.53) | <0.001^a^ | -7.002 | -3.888 |
| Temp (°C) | Home vs. Control | -5.41 (1.14) | 0.003^a^ | -8.747 | -2.063 |
|  | Home vs. ICU | -5.39 (1.62) | 0.027^a^ | -10.137 | -0.633 |
|  | Control vs. ICU | 0.02 (0.71) | 1.000 | -2.067 | 2.107 |
| TST (min) | Home vs. Control | -4.90 (14.96) | 1.000 | -48.781 | 38.981 |
|  | Home vs. ICU | 42.75 (14.35) | 0.046^a^ | 0.656 | 84.844 |
|  | Control vs. ICU | 47.65 (10.39) | 0.004^a^ | 17.160 | 78.140 |
| Sleep efficiency (%) | Home vs. Control | 3.17 (2.92) | 0.919 | -5.395 | 11.726 |
|  | Home vs. ICU | 6.15 (3.62) | 0.370 | -4.468 | 16.775 |
|  | Control vs. ICU | 2.99 (3.05) | 1.000 | -5.968 | 11.943 |
| Sleep latency (min) | Home vs. Control | -7.33 (15.61) | 1.000 | -53,127 | 38.470 |
|  | Home vs. ICU | -13.73 (12.13) | 0.861 | -49.315 | 21.861 |
|  | Control vs. ICU | -6.40 (13.37) | 1.000 | -45.626 | 32.829 |
| REM latency (min) | Home vs. Control | -1.45 (21.98) | 1.000 | -65.926 | 63.026 |
|  | Home vs. ICU | -46.90 (8.49) | 0.001^a^ | -71.799 | -22.001 |
|  | Control vs. ICU | -45.45 (23.29) | 0.248 | -113.758 | 22.858 |
| REM (%) | Home vs. Control | -1.69 (1.57) | 0.934 | -6.307 | 2.929 |
|  | Home vs. ICU | 2.88 (1.86) | 0.465 | -2.563 | 8.328 |
|  | Control vs. ICU | 4.57 (1.75) | 0.086 | -0.576 | 9.719 |
| N1 (%) | Home vs. Control | -0.63 (0.61) | 0.986 | -2.410 | 1.155 |
|  | Home vs. ICU | -1.44 (0.80) | 0.316 | -3.800 | 0.910 |
|  | Control vs. ICU | -0.82 (1.05) | 1.000 | -3.898 | 2.263 |
| N2 (%) | Home vs. Control | 0.00 (1.01) | 1.000 | -2.967 | 2.954 |
|  | Home vs. ICU | -8.00 (2.05) | 0.011^a^ | -14.012 | -1.970 |
|  | Control vs. ICU | -8.00 (1.69) | 0.003^a^ | -12.932 | -3.037 |
| N3 (%) | Home vs. Control | 2.33 (2.39) | 1.000 | -4.678 | 9.338 |
|  | Home vs. ICU | 6.56 (2.41) | 0.070 | -0.498 | 13.619 |
|  | Control vs. ICU | 4.23 (1.84) | 0.140 | -1.155 | 9.616 |
| WASO (%) | Home vs. Control | -7.05 (7.68) | 1.000 | -29.579 | 15.479 |
|  | Home vs. ICU | -47.15 (17.90) | 0.082 | -99.655 | 5.355 |
|  | Control vs. ICU | -40.10 (15.99) | 0.100 | -86.989 | 6.789 |
| Awakenings per night | Home vs. Control | -2.25 (2.81) | 1.000 | -10.495 | 5.995 |
|  | Home vs. ICU | -6.95 (4.00) | 0.350 | -18.696 | 4.796 |
|  | Control vs. ICU | -4.70 (2.44) | 0.259 | -11.859 | 2.459 |
| Mean duration of awakenings (min) | Home vs. Control | -0.07 (0.09) | 1.000 | -0.345 | 0.203 |
|  | Home vs. ICU | -0.71 (0.25) | 0.062 | -1.459 | 0.035 |
|  | Control vs. ICU | -0.64 (0.23) | 0.060 | -1.307 | 0.025 |
| Arousal index | Home vs. Control | -3.70 (2.20) | 0.379 | -10.150 | 2.745 |
|  | Home vs. ICU | -8.98 (2.10) | 0.006^a^ | -15.126 | -2.826 |
|  | Control vs. ICU | -5.27 (2.24) | 0.130 | -11.852 | 1.305 |
| RR_arousal_ | Home vs. Control | -8.17 (1.68) | 0.003^a^ | -13.108 | -3.235 |
|  | Home vs. ICU | -0.37 (0.18) | 0.219 | -0.901 | 0.164 |
|  | Control vs. ICU | 7.80 (1.72) | 0.004^a^ | 2.755 | 12.850 |

LAeq = A-weighted per second sound level, TST = Total sleep time, REM = Rapid eye movement sleep, RR_arousal_ = Relative risk of arousal after ΔdB>6, WASO = Wake time after sleep onset

^a^Significant p values are highlighted

Bonferroni adjusted pairwise comparison, with numbers based on estimated marginal means. Data are presented as the mean (SD).
